# The relationship between baseline Organizational Readiness to Change Assessment subscale scores and implementation of hepatitis prevention services in substance use disorders treatment clinics: a case study

**DOI:** 10.1186/1748-5908-5-46

**Published:** 2010-06-14

**Authors:** Hildi J Hagedorn, Paul W Heideman

**Affiliations:** 1 Substance Use Disorders Quality Enhancement Research Initiative, Minneapolis VA Medical Center, Minneapolis, MN, USA; 2 Department of Psychiatry, School of Medicine, University of Minnesota, Minneapolis, MN, USA; 3 Hepatitis C Resource Center, Minneapolis VA Medical Center, Minneapolis, MN, USA; 4 Minneapolis VA Medical Center, Minneapolis, MN, USA

## Abstract

**Background:**

The Organizational Readiness to Change Assessment (ORCA) is a measure of organizational readiness for implementing practice change in healthcare settings that is organized based on the core elements and sub-elements of the Promoting Action on Research Implementation in Health Services (PARIHS) framework. General support for the reliability and factor structure of the ORCA has been reported. However, no published study has examined the utility of the ORCA in a clinical setting. The purpose of the current study was to examine the relationship between baseline ORCA scores and implementation of hepatitis prevention services in substance use disorders (SUD) clinics.

**Methods:**

Nine clinic teams from Veterans Health Administration SUD clinics across the United States participated in a six-month training program to promote evidence-based practices for hepatitis prevention. A representative from each team completed the ORCA evidence and context subscales at baseline.

**Results:**

Eight of nine clinics reported implementation of at least one new hepatitis prevention practice after completing the six-month training program. Clinic teams were categorized by level of implementation-high (n = 4) versus low (n = 5)-based on how many hepatitis prevention practices were integrated into their clinics after completing the training program. High implementation teams had significantly higher scores on the patient experience and leadership culture subscales of the ORCA compared to low implementation teams. While not reaching significance in this small sample, high implementation clinics also had higher scores on the research, clinical experience, staff culture, leadership behavior, and measurement subscales as compared to low implementation clinics.

**Conclusions:**

The results of this study suggest that the ORCA was able to measure differences in organizational factors at baseline between clinics that reported high and low implementation of practice recommendations at follow-up. This supports the use of the ORCA to describe factors related to implementing practice recommendations in clinical settings. Future research utilizing larger sample sizes will be essential to support these preliminary findings.

## Background

Experts in organizational change contend that organizational readiness to change is critical to successful implementation of new practices [1-5]. However, as pointed out in a review by Weiner *et al*. [6], health services researchers have only just begun theorizing about and developing measures of organizational readiness to change. Weiner and colleagues reviewed the conceptualization and measurement of organizational readiness to change, drawing from not only health services research but also business, education, and human services journals. The basic conclusions of the review were that there is little consistency in conceptual terminology regarding organizational readiness to change, and most currently available instruments for measuring the construct have limited evidence of reliability and validity. Particularly lacking is evidence that measures of organizational readiness to change can predict organizational-level outcomes. The authors cite only four such studies [7-10], with three being from the business rather than the healthcare sector. These studies used surveys that assessed readiness to change and outcomes simultaneously. None of these studies examined whether organizational readiness to change is related to actual future implementation of new practices. Two additional recent studies assessed the ability of an organizational readiness to change measure to predict organization-level outcomes [11,12]; one retrospectively assessed non-adoption of a new technology [11], and the other offered a qualitative description of the relationship between organizational readiness to change and implementation outcomes [12]. In healthcare, with its current heavy focus on interventions designed to implement evidence-based practices, what is sorely needed is a measure that demonstrates a correlation to actual uptake of new practices following such interventions. Such a measure could provide insight into the likelihood of successful implementation within a particular site prior to the investment of resources or could allow tailoring of an implementation intervention to the specific needs of participating sites. In addition to the deficiencies in the construct definition and measurement of organizational readiness to change reported by Weiner *et al*. [6], the lack of evidence supporting the correlation of measures of organizational readiness to change with implementation intervention outcomes can be added to the list of deficiencies in the field as it currently stands.

### The Organizational Readiness to Change Assessment (ORCA)

The ORCA is an instrument that was developed by the Veterans Administration's (VA) Ischemic Heart Disease Quality Enhancement Research Initiative for assessing organizational readiness for implementation of evidence-based healthcare interventions [13,14]. A significant strength of the ORCA is that it was developed based on the Promoting Action on Research Implementation in Health Services (PARIHS) model, a conceptual framework that has shown promise in guiding implementation efforts in healthcare [15-17]. Helfrich and colleagues examined the psychometrics of the ORCA using cross-sectional data from three quality improvement projects conducted in VA medical centers [14]. Psychometric analyses indicated general support for the reliability of ORCA items and for the three primary scales of the ORCA [14]. Factor analyses supported a three-factor structure as hypothesized by the PARIHS framework. In addition to being model-driven and having promising psychometric properties, the ORCA fits many of the recommendations found in the Weiner review regarding defining and measuring the construct of organizational readiness to change [6]. The ORCA measures readiness to change at the organizational level and focuses the respondent on a specific change referent rather than innovation in general. It is designed to be used after an organization has agreed to adopt a change but prior to the start of implementation efforts. The ORCA assesses aspects of both willingness of respondents to adopt the new practice (*e.g.*, agreement with the evidence, innovative culture) and capability to implement change (*e.g.*, available resources, leadership effectiveness). The ORCA measures not only whether resources are available to the organization, but whether the respondents perceive that those resources will be made available for the intended change.

For these reasons, the ORCA seems to have potential as a robust measure of organizational readiness to change. However, no research to date has addressed the predictive validity of the ORCA scales. Specifically, it is not known whether the ORCA scales predict outcomes in implementation projects.

### The current study

The present study aimed to assess whether higher levels of pre-training organizational readiness, operationalized as higher scores on the ORCA scales, were related to greater implementation of hepatitis prevention practices following completion of the Liver Health Initiative (LHI) training program.

## Methods

Teams from VA substance use disorders (SUD) clinics voluntarily enrolled in the 2007 LHI training program by responding to advertisements on the VA national addictions email group and on the VA quarterly SUD national conference calls. The advertisements stated that the clinical team should include a member of the SUD clinic leadership (*i.e.*, medical director, program coordinator, chief nurse, *et al*.), one frontline SUD provider interested in integrating hepatitis services into SUD treatment, and one frontline hepatitis clinician.

Overall, 11 clinic teams from across the United States responded and were enrolled in the LHI training program. Two of the clinic teams did not provide follow-up data on implementation outcomes and therefore were excluded from the current study. The sample for the current study includes nine teams. One of the teams did not provide baseline ORCA context scale responses so comparisons of context scale scores to implementation outcomes include only eight teams. Clinic demographics, the ORCA, and baseline implementation of hepatitis prevention services were collected by paper and pencil survey from each team leader six weeks prior to attending the face-to-face portion of the training. Team demographics were collected from registration and attendance records for the face-to-face portion of the training. Facility demographics were collected from VA public databases. Implementation outcomes were collected by paper and pencil survey from each team leader one, three, and six months after completing the face-to-face portion of the training program.

### Overview of the LHI

The LHI is sponsored by the VA SUD Quality Enhancement Research Initiative and Hepatitis C Resource Center. The goal of the LHI is to improve prevention, identification, and treatment of hepatitis among patients seeking treatment at VA SUD clinics. Specific goals of the LHI are based on a successful Healthy Liver program established in the Minneapolis VA Medical Center's Addictive Disorders Service [18]. The program trains substance use disorder clinics to provide: testing for hepatitis B and C and immunities to hepatitis A and B; comprehensive patient education on hepatitis infections and liver health; hepatitis A and B immunizations; and expedited referrals to hepatitis treatment providers for patients diagnosed with hepatitis B or hepatitis C. Teams participating in the LHI training program complete a baseline needs assessment that provides the team with a basis for the later development of their action plan. Teams then attend a 1.5-day training at the Minneapolis VA Medical Center. The first day of training provides information on the risks for and impact of liver disease in patients with SUD, the goals of the LHI, and a quality improvement process for implementing the LHI goals. The remaining half-day of the training assists teams in development of action plans facilitated via an Action Plan form that lists the recommendations of the LHI and asks teams to identify specific improvement goals and action steps. Action plans are then presented to the rest of the trainees and the program faculty for feedback. To support progress on improvement goals and actions steps, external facilitation is provided via telephone for six months.

## Measures

### Team, clinic, and facility demographics

Team information included the number of team members attending and their job titles. Clinic demographics included the number of full-time equivalent staff members in the SUD clinic, the average number of new patient intakes completed each month, and the total number of current patients receiving services at the clinic.

Facility demographics included facility type (medical center versus community-based outpatient clinic) and facility complexity level. Every VA medical center is assigned to a complexity level group based on the VA's 2005 Facility Complexity Model. The model employs several variables, including the total number of patients served by the facility, the number and types of intensive care units in the facility, the number of resident programs and the total number of resident slots available, the total amount of research dollars managed by a facility, and the number and breath of physician specialists employed by the facility. The model uses a hierarchical clustering method to assign each medical center to a group. This method of grouping hospitals was based on work by Bazzoli and her colleagues [19,20] but the specific variables used for grouping hospitals were revised to maximize relevance specifically to VA medical centers. Based on the algorithm, each medical center receives a score of 1 (high complexity), 2 (medium complexity), or 3 (low complexity). Because one-half of all VA medical centers are high complexity, the group receiving scores of 1 is further divided into sub-categories of 1A, 1B, and 1C, with 1A representing the highest level of complexity followed by 1B and 1C.

### Organizational Readiness to Change Assessment (ORCA)

The ORCA is a 77-item scale designed to measure the elements and sub-elements of the PARIHS model that are theorized to be related to successful implementation outcomes. The ORCA consists of three scales corresponding to the three PARIHS model primary elements: strength and extent of evidence for clinical practice changes [21]; quality of the organizational context [22]; and capacity for internal facilitation [23]. The evidence scale consists of four subscales. The first subscale is comprised of two items that assess the discrepancy between the respondent's opinion of the strength of evidence base and the opinion of their colleagues. The remaining three subscales are research evidence, clinical experience, and patient preferences, reflecting sub-elements of the PARIHS evidence element. The context scale contains six subscales: two assess aspects of organizational culture (leadership culture and staff culture); one represents leadership practices; one assesses measurement (*e.g.*, leadership feedback); one assesses readiness to change among opinion leaders; and one subscale examines resources to support general practice changes. Capacity for internal facilitation has nine subscales: two examining senior leadership characteristics; five examining implementation of various organization characteristics such as planning and progress; one measuring clinical champion characteristics such as carrying out a project; and one measuring communication. See Table 1 for the ORCA items corresponding to each scale and subscale. Each sub-scale consists of three to six items. All items are scored on a 1 to 5 Likert scale with anchors of 1 = strongly disagree and 5 = strongly agree. Scale and subscale scores are calculated by dividing the total score by the number of items on the scale resulting in scale score values of 1 to 5.

**Table 1:** Organizational readiness to change assessment items

Scale	Sub-Scale	Items
Evidence*	Research	The proposed practice changes or guideline implementation:
		Are(is) supported by RCTs or other scientific evidence from the VA.
		Are(is) supported by RCTs or other scientific evidence from other healthcare systems.
		Should be effective, based on current scientific knowledge.
		Are(is) experimental, but may improve patient outcomes.
		Likely won't make much difference in patient outcomes**.

	Clinical Experiences	The proposed practice changes or guideline implementation:
		Are supported by clinical experience with VA patients.
		Are supported by clinical experience with patients in other healthcare systems.
		Conform to the opinions of clinical experts in this setting.
		Have not been attempted in this setting**.

	Patient Preferences	The proposed practice changes or guideline implementation:
		Have been well-accepted by VA patients in a pilot study.
		Are consistent with clinical practices that have been accepted by VA patients.
		Take into consideration the needs and preferences of VA patients.
		Appear to have more advantages than disadvantages for VA patients.

Context	Leadership Culture	Senior leadership/clinical management in your organization:
		Reward clinical innovation and creativity to improve patient care.
		Solicit opinions of clinical staff regarding decisions about patient care.
		Seek ways to improve patient education and increase patient participation in treatment.

	Staff Culture	Staff members in your organization:
		Have a sense of personal responsibility for improving patient care and outcomes.
		Cooperate to maintain and improve effectiveness of patient care.
		Are willing to innovate and/or experiment to improve clinical procedures.
		Are receptive to change in clinical processes.

	Leadership	Senior leadership/clinical management in your organization:
		Provide effective management for continuous improvement of patient care.
		Clearly define areas of responsibility and authority for clinical managers and staff.
		Promote team building to solve clinical care problems.
		Promote communication among clinical services and units.

	Measurement	Senior leadership/clinical management in your organization:
		Provide staff with information on VA performance measures and guidelines.
		Establish clear goals for patient care processes and outcomes.
		Provide staff members with feedback/data on effects of clinical decisions.
		Hold staff members accountable for achieving results.

	Opinion Leaders	Opinion leaders in your organization:
		Believe that the current practice patterns can be improved.
		Encourage and support changes in practice patterns to improve patient care.
		Are willing to try new clinical protocols.
		Work cooperatively with senior leadership/clinical management to make appropriate changes.

	Resources	In general in my organization, when there is agreement that change needs to happen:
		We have the necessary support in terms of budget or financial resources.
		We have the necessary support in terms of training.
		We have the necessary support in terms of facilities.
		We have the necessary support in terms of staffing.

Facilitation	Leaders' Practices	Senior leadership/clinical management will:
		Propose a project that is appropriate and feasible.
		Provide clear goals for improvement in patient care.
		Establish a project schedule and deliverables.
		Designate a clinical champion(s) for the project.

	Clinical Champion	The project clinical champion:
		Accepts responsibility for the success of this project.
		Has the authority to carry out the implementation.
		Is considered a clinical opinion leader.
		Works well with the intervention team and providers.

	Leadership Implementation Roles	Senior leadership/clinical management/staff opinion leaders:
		Agree on the goals for this intervention.
		Will be informed and involved in the intervention.
		Agree on adequate resources to accomplish the intervention.
		Set a high priority on the success of the intervention.

	Implementation Team Roles	The implementation team members:
		Share responsibility for the success of this project.
		Have clearly defined roles and responsibilities.
		Have release time or can accomplish intervention tasks within their regular work load.
		Have staff support and other resources required for the project.

	Implementation Plan	The implementation plan for this intervention:
		Identifies specific roles and responsibilities.
		Clearly describes tasks and timelines.
		Includes appropriate provider/patient education.
		Acknowledges staff input and opinions.

	Project Communication	Communication will be maintained through:
		Regular project meetings with the project champion and team members.
		Involvement of quality management staff in project planning and implementation.
		Regular feedback to clinical management on progress of project activities and resource needs.
		Regular feedback to clinicians on effects of practice changes on patient care/outcomes.

	Project Progress Tracking	Progress of the project will be measured by:
		Collecting feedback from patients regarding proposed/implemented changes.
		Collecting feedback from staff regarding proposed/implemented changes.
		Developing and distributing regular performance measures to clinical staff.
		Providing a forum for presentation/discussion of results and implications for continued improvements.

	Project Resources	The following are available to make the selected plan work:
		Staff incentives.
		Equipment and materials.
		Patient awareness/need.
		Provider buy-in.
		Intervention team.
		Evaluation protocol.

	Project Evaluation	Plans for evaluation and improvement of this intervention include:
		Periodic outcome measurement.
		Staff participation/satisfaction survey.
		Patient satisfaction survey.
		Dissemination plan for performance measures.
		Review of results by clinical leadership.

* The Evidence questions are preceded by a statement identifying the evidence-based practice that is the current target of intervention.

** Denotes items that are reverse coded.

Team leaders completed the ORCA six weeks prior to attending the face-to-face portion of the training program. For the purpose of this study, only the evidence and context scale items were completed by the team leaders. The current study did not examine the facilitation scale because the items in that scale assess material that was not applicable before completing the 1.5-day training and developing an action plan (*e.g.*, questions regarding the implementation plan, the role of the implementation team, *et al*.).

### Assessment of baseline implementation and implementation outcomes

At baseline (six weeks prior to the face-to-face portion of the training program) and at one, three, and six months after completing the face-to-face training, team leaders completed a survey evaluating their clinic's current practices related to hepatitis screening, education, prevention, and treatment referral. Questions on the survey asked team leaders to report whether their clinic provided routine hepatitis B and C testing to new clients and whether their clinic provided routine testing for immunity to hepatitis A and B. If testing was provided in the clinics, team leaders were asked to report what procedures were in place to provide feedback of test results to clients and what procedures were in place to refer clients who tested positive for hepatitis B or C for care. Team leaders were also asked whether vaccinations for hepatitis A and B were available in their clinic. Finally, they were asked to report whether they provided education regarding hepatitis infections in their clinic and if they did, which clients were targeted for this education (*e.g.*, all or only those in a specific program such as an intensive outpatient program or a methadone program). The LHI training program specifically recommends eight practices for hepatitis prevention and care in SUD clinics, which are summarized in Appendix 1. The team leaders' survey responses were compared to the eight LHI practice recommendations, and the clinic received one point for each practice that was currently in place. 'Implementation scores' could therefore range from 0 to 8 depending on current clinic practices, with higher scores reflecting greater implementation of recommended practices.

Team leaders from all nine clinics completed the baseline survey. Team leaders from eight clinics completed the one-month survey. Three-month surveys were also completed by eight team leaders. Only four team leaders completed the six-month survey. A decision was made to use the latest follow-up available for each clinic as the best assessment of their final state of implementation progress. Therefore, implementation outcome was based on one-month follow-up data for one clinic, three-month data for four clinics, and six-month data for four clinics.

Based on a median split of implementation scores at the final follow-up, clinics who reported utilizing at least six of the eight recommended practices were classified as having high implementation of LHI recommendations (n = 4) and those who reported 5 or fewer practices were classified as having low implementation of LHI recommendations (n = 5).

### Data analysis

Mean ORCA subscale scores were calculated for high implementation clinics (defined as clinics receiving an implementation score of 6 or greater at the final follow-up available) and low implementation clinics (defined as clinics receiving an implementation score of 5 or less at the final follow-up available). Because of the small sample size, the magnitude of the difference in mean ORCA subscale scores between high and low implementation clinics was evaluated using effect sizes (Cohen's *d*) and 95% confidence intervals rather than employing tests for statistically significant differences.

## Results

### Demographics

See Table 2 for specific clinic demographics. The nine participating SUD clinic teams ranged in size from one to four members. Team members included five nurse practitioners, four clinical nurse specialists, three registered nurses, two clinical social workers, one physician, one addiction therapist, and one physicians' assistant. Of the nine clinic teams, eight were from SUD clinics that resided within VA medical centers and one was from a VA community-based outpatient clinic. Of the eight teams from medical centers, five came from high complexity medical centers, two came from medium complexity centers, and one came from a low complexity center. The number of full-time equivalent staff assigned to each clinic ranged from 4.5 to 21. The average number of new patients served each month ranged from 3 to 80. The total number of patients currently receiving services ranged from 40 to 447.

**Table 2:** Team/clinic demographics and baseline and follow-up implementation scores

Site ID	Team Members*	Facility Type	Complexity Level***	Staffing (FTE) †	Intakes per Month ††	Total Patient Census †††	Implementation Scores				Final Implementation Category
								
							Baseline	Month 1	Month 3	Month 6	
1	CNS	CBOC**	N/A	4.5	3	58	4	6	7	- (missing data)	High

2	RN	Medical Center	1A	5.5	10	-	1	-	3	-	Low

3	CSW, CNS, PA, NP	Medical Center	1B	20.5	8	447	2	4	4	5	Low

4	NP, RN	Medical Center	1A	20	80	65	7	6	8	-	High

5	NP, MD	Medical Center	1C	8	-	165	2	6	6	6	High

6	CSW	Medical Center	2	21	30	227	3	4	-	-	Low

7	CNS, RN	Medical Center	3	6	-	-	5	5	6	5	Low

8	AT, NP	Medical Center	2	11	40	40	6	7	7	-	High

9	CNS, NP	Medical Center	1B	9	40	142	2	2	2	3	Low

*CNS = Clinical Nurse Specialist; RN = Registered Nurse; CSW = Clinical Social Worker; PA = Physician's Assistant; NP = Nurse Practitioner; MD = Medical Doctor; AT = Addiction Therapist

**CBOC = Community-Based Outpatient Clinic

*** 1 = High Complexity (A representing highest level in this category followed by B and C); 2 = Medium Complexity; 3 = Low Complexity

† FTE = Full-Time Equivalent

†† Average number of new patients seen in one month.

††† Total number of patients receiving any services from the clinic at the time of baseline.

### Implementation of LHI recommendations

Refer to Table 2 for implementation scores at baseline and follow-up points for each clinic. At baseline (n = 9), implementation of the eight LHI recommendations ranged from 1 to 7 (M = 3.67). At one-month follow-up (n = 8), implementation scores ranged from 2 to 7 (M = 5). At three-month follow-up (n = 8), implementation scores ranged from 2 to 8 (M = 5.38). At six-month follow-up (n = 4), implementation scores ranged from 3 to 6 (M = 4.75). Final implementation scores, based on the latest follow-up information available for each clinic, ranged from 3 to 8 (M = 5). Implementation of new services from baseline to follow-up ranged from 0 (one clinic only) to 4 (M = 1.78).

### ORCA Responses

After the clinics were divided into the low and high implementation groups, their responses to the ORCA administered at baseline were compared. See Table 3 for descriptive data and effect sizes for ORCA subscales for high and low implementation groups. ORCA scores on patient preferences and leadership culture subscales were significantly higher (95% confidence interval does not include 0) for high implementation clinics compared to low implementation clinics. Differences between high and low implementation clinics on other ORCA subscales did not reach statistical significance in this small sample size. However, large effect sizes (Cohen's *d *>0.80) in the hypothesized direction were found for the research, clinical experience, staff culture, and leadership behavior subscales. A medium effect size (Cohen's *d *= 0.61) in the hypothesized direction was found for the measurement subscale. The opinion leaders subscale did not appear to be related to implementation scores. Contrary to hypotheses, low implementation clinics reported greater scores on the General Resources subscale compared to high implementation clinics (Cohen's *d *= -0.94).

**Table 3:** Descriptive data and effect sizes of ORCA responses for high and low implementation clinics

ORCA Subscale	n	Mean (SD)	Effect size (d), (95% CI)
**Evidence Scale**			

Research			

low	5	4.00 (0.28)	1.01 (-0.48 - 2.27)

high	4	4.25 (0.19)	

Clinical Experience			

low	5	3.25 (1.30)	1.26 (-0.29 - 2.54)

high	4	4.56 (0.52)	

Patient Preferences			

low	5	3.40 (0.45)	2.15 (0.33 - 3.50)

high	4	4.50 (0.58)	

**Context Scale**			

Leader Culture			

low	5	3.87 (0.18)	2.09 (0.13 - 3.50)

high	3	4.56 (0.51)	

Staff Culture			

low	5	4.10 (0.82)	.82 (-0.76 - 2.19)

high	3	4.67 (0.29)	

Leadership Behavior			

low	5	3.65 (0.58)	1.12 (-0.54 - 2.48)

high	3	4.08 (0.14)	

Measurement			

low	5	4.15 (0.60)	0.61 (-0.92 - 1.99)

high	3	4.50 (0.50)	

Opinion Leaders			

low	5	4.30 (0.69)	-0.18 (-1.59 - 1.27)

high	3	4.17 (0.76)	

General Resources			

low	5	3.55 (1.04)	-0.94 (-2.31 - 0.67)

high	3	3.08 (0.76)	

## Discussion

The purpose of this study was to assess the relationship between baseline ORCA evidence and context subscales and implementation of practice recommendations following a training experience. Results indicated differences in the hypothesized direction between high and low implementation clinics on several ORCA subscales. The relationship between ORCA subscale scores and implementation outcomes does not appear to be related to the number of team members that were sent to the face-to-face training, the type of facility or complexity of the facility the team came from, or the number of staff employed by or number of patients served by the SUD clinic. High implementation teams included both a 'team' of one clinical nurse specialist from a community-based outpatient clinic with only 4.5 full-time equivalent staff members as well as a team from a medical center with the highest possible complexity rating and 20 full-time equivalent staff members. On the other hand, the low implementation teams included both a team from a low complexity medical center with only six full-time equivalent staff members and a team from a high complexity medical center with over 20 full-time equivalent staff members. It would appear that the ORCA subscales are capturing something about the organization's readiness to implement practice change related to hepatitis prevention that is not fully explained by the size or complexity of the SUD clinic itself or the medical center in which it resides.

The relationship of the patient preferences and leadership culture subscales to implementation of recommended practices were particularly robust, with effect sizes of 2.15 and 2.09, respectively. Based on the items in the patient preference subscales, it appears that team leaders who more strongly endorsed the idea that hepatitis services provided through the SUD treatment clinics would be accepted by patients and meet patients' needs and expectations were associated with clinics that implemented more recommended practices. Similarly, based on the items in the leadership culture subscale, it appears that team leaders who more strongly endorsed that their clinic leadership provided effective management, clearly defined staff responsibilities, and promoted team building and communication were associated with clinics that implemented more recommended practices. These findings support the hypotheses from the PARIHS model that a match between perceived patient needs and the new practice to be implemented and effective leadership facilitate implementation of new practices [17].

the opinion leaders subscale was the only subscale to yield a small effect size when comparing low and high implementation clinics. This result may be explained by the recruitment method for this study. All teams volunteered to participate, which resulted in a sample of team members presumably eager to make improvements to their healthcare practices. Support for this contention is found when examining the means for high and low implementation clinics on this subscale. For high implementation clinics, the opinion leaders subscale score fell in a similar range to other subscales, whereas for low implementation clinics the opinion leader score was the highest subscale score, closer to the subscale scores of the high implementation clinics. Perhaps the low implementation team leaders felt they had supportive opinion leaders within their team but recognized that other facilitators of change were lacking in their organization.

The only subscale that did not function in the hypothesized direction was the resource subscale. This scale yielded a large effect size, with low implementation clinics reporting greater resources (*e.g.*, financial, facilities, training) for change than high implementation clinics. Generally speaking, slack resources are viewed as a facilitator for implementation. However, as Wiener and colleagues pointed out in their discussion of the definition of the construct of organizational readiness to change, an organization may have all of the necessary financial and material resources to implement a change but lack the motivation or the capability to mobilize those resources [6]. The resource subscale questions on the ORCA begin with the stem, 'In general in my organization, when there is an *agreement that change needs to happen...*'. Given that the evidence subscales indicate that the team leaders from the low implementation sites expressed lower levels of support for the LHI recommendations, they may very well feel that the resources are available to them but they have not committed to mobilizing those resources to implement these particular recommendations. Another potential hypothesis is that team leaders from the low implementation sites may have had less experience with the level of resources necessary to implement a new practice and therefore may overestimate the adequacy of available resources. This could potentially lead to discouragement when initial attempts to implement practice change are unsuccessful or run into significant barriers. Interestingly, the resources subscale was also problematic when Helfrich *et al*. investigated the factor structure of the ORCA in that it did not load onto the context scale as predicted [14]. Nor did it significantly load on either of the other primary scales of the ORCA. Instead, it appears to measure information separate from the evidence, context, and facilitation scales. While a certain minimum level of slack resources is presumably necessary for successful implementation, it does not appear to be sufficient, because mobilization of those resources may be dependent on perceived need for change and the capabilities of the implementation team to capitalize on those available resources.

## Lessons learned

In addition to providing preliminary support for the use of the ORCA as a baseline measure of organizational readiness to change, the experience using the ORCA in an implementation study has led to some recommendations for others who may wish to use it in this capacity. Having only the team leader complete the measure limits its reliability, so during a subsequent LHI training program we requested that team leaders distribute the ORCA to all clinic staff for completion. This was also a relatively unsuccessful strategy because many of clinic staff members were not involved in the implementation project and so were confused by the questions. Response rates with this strategy were quite low. For future training programs, we intend to more strictly enforce the requirement of a minimum of three team members per implementation site and to administer the ORCA to all implementation team members. Second, we are now planning to change the timing of the completion of the ORCA from prior to the face-to-face training to immediately following the face-to-face training. This change in timing will still measure organizational readiness to change prior to the start of any implementation activities by the team. However, the change in timing will have the advantage of providing the team members with a better understand of exactly what they are expected to do, allowing us to take advantage of the facilitation scale, which includes questions which did not make sense to team leaders prior to the face-to-face training. Finally, the new timing takes advantage of the 'captive audience' because they will be required to complete the ORCA as the final portion of the face-to-face training. In the future, we plan to use the ORCA scales to attempt to identify sites potentially at risk for poor implementation outcomes and to target those sites for more intensive external facilitation in an effort to improve the overall outcomes of the LHI training program.

## Limitations

Given the exploratory nature of this study, there are limitations to the findings. First, this study included only nine clinics and only collected ORCA information from the team leader from each clinic. This limits the reliability of the ORCA because it is limited to the perspective of one person from the clinic. Granted, the perspective of the team leader may be the most important for predicting successful practice change, but gathering ORCA data from a broader sample of clinic staff would presumably increase the reliability of the data. The small sample size limits the generalizability of the findings and tempers the confidence that can be placed in the results. However, the sample did include medical centers representing the full range of complexity scores as well as one community-based outpatient clinic. The SUD clinics also ranged from very small to very large. Additional research using the ORCA to measure implementation of clinical practices within organizations needs to be completed before any firm conclusions can be made.

A second limitation is that this study sampled volunteer clinics. This suggests that these clinics were already motivated to improve healthcare practices before attending the LHI training. Ongoing research includes clinics that are mandated to attend training, which will allow a comparison of ORCA scores and outcomes for volunteer versus mandated attendees. In addition, two clinics would have been classified as highly adherent to LHI recommendations prior to training, making it difficult to distinguish whether the ORCA was assessing ability to implement new practices or was simply correlated with baseline clinic functioning. We considered using change in implementation score from baseline to follow-up rather than only follow-up implementation score as the criteria for separating high and low implementation clinics. However, this would have classified the two clinics with high baseline implementation scores as low implementers, suggesting that they were somehow deficient. In fact, these two clinics were able to implement innovative practices available in few VA SUD clinics prior to their participation in a formal implementation intervention and both were able to add one additional intervention during their participation in the LHI. While these two clinics differ from clinics that moved from low to high implementation during the intervention period, we felt they had more in common with the high rather than the low implementers. In the future, low implementation of recommended practices at baseline should serve as a screening criterion for training. This would allow the follow-up implementation measure to function as a truer measure of change and would also focus resources on clinics in greatest need of the LHI training.

Finally, the labeling of the clinics as high and low implementation was somewhat arbitrary. The median split based on the number of clinical practices reported at follow-up labeled clinics with five or fewer of the eight practices as low, while clinics reporting six or more of the eight practices were labeled as high. One could argue that a clinic utilizing at least five of eight recommended clinical practices could also be identified as a high implementation clinic. However, some of the LHI recommendations could be considered 'low hanging fruit.' For example, if a clinic is already doing routine laboratory testing at intake, adding hepatitis B and C testing to the routine testing panel may be a relatively easy change in practice compared to negotiating the availability and administration of hepatitis A and B vaccinations in the clinic. Setting a high bar for the high implementation category required that teams that achieved this category had implemented at least two of the more difficult recommendations. Future studies with larger sample sizes may wish to utilize regression statistics to avoid the need to categorize clinics.

Given the limitations of this study, most particularly the small sample size, we consider this study to be preliminary. We do not consider it to provide definitive support for the use of the ORCA as a predictor of implementation success but rather hope that our experience, our mistakes, and what we have learned from them will assist in the development of more rigorous future research.

## Summary

This exploratory study was the first to examine the utility of the ORCA as a baseline measure of organizational readiness to change and to examine the relationship of the ORCA to implementation of practice recommendations following an intervention. The results provide preliminary support for the use of the ORCA to measure organizational factors that appear to influence the success of implementation. The results also suggest that the ORCA is measuring factors independent of the size and/or complexity of the organization. Despite the obvious limitations of a study with this small sample size, at the present time there is a scarcity of published work relating any organizational readiness to change measure to specific recommended implementation outcomes at the organizational level. This is likely because of the significant challenges faced when undertaking work of this type. Sample sizes are limited by the fact that the organization is the unit of interest and by attrition from baseline through the implementation and follow-up phase. The outcome of 'successful implementation' is often subjective and difficult to operationalize and quantify. Larger sample sizes to provide a more definitive assessment of the validity of the ORCA will likely require the collating of results from multiple implementation studies with the added complication that outcome measures will differ by study depending on the practice change that is recommended. The VA has recently funded such an effort, and results from that effort will likely be available in two years.

## Competing interests

The authors declare that they have no competing interests.

## Authors' contributions

HJH developed the LHI and the evaluation measures in collaboration with the Minneapolis Hepatitis C Resource Center team. PWH and HJH conceived of the study and participated in its design. PWH completed the data analysis and wrote the first draft of the manuscript. HJH wrote subsequent versions of the manuscript. All authors participated in the drafting of the manuscript and read and approved it in its final form.

## Appendix 1. Summary of Liver Health Initiative recommended practices

Routine testing of all new patients for hepatitis C.

Routine testing of all new patients for hepatitis B.

Routine testing of all new patients for immunity to hepatitis A.

Routine testing of all new patients for immunity to hepatitis B.

Standardized procedures in place for communicating test results to all tested patients.

Referral of all hepatitis B and C positive patients to specialty care for follow-up.

Availability of hepatitis B and C vaccinations in the substance use disorders clinic.

Standardized, comprehensive education on hepatitis infections offered to all patients as part of routine care.
